# Crossed Pathways for Radiation-Induced and Immunotherapy-Related Lung Injury

**DOI:** 10.3389/fimmu.2021.774807

**Published:** 2021-12-01

**Authors:** Zengfu Zhang, Jialin Zhou, Vivek Verma, Xu Liu, Meng Wu, Jinming Yu, Dawei Chen

**Affiliations:** ^1^ Department of Radiation Oncology, Cheeloo College of Medicine, Shandong University, Jinan, China; ^2^ Department of Radiation Oncology, Laboratory of Radio-Immunology, Cancer Research Center, Shandong Cancer Hospital and Institute, Shandong First Medical University and Shandong Academy of Medical Sciences, Jinan, China; ^3^ Department of Experimental Radiation Oncology, The University of Texas MD Anderson Cancer Center, Houston, TX, United States

**Keywords:** radiation-induced lung injury, immunotherapy-related lung injury, immune checkpoint inhibitor, signaling pathway, pyroptosis

## Abstract

Radiation-induced lung injury (RILI) is a form of radiation damage to normal lung tissue caused by radiotherapy (RT) for thoracic cancers, which is most commonly comprised of radiation pneumonitis (RP) and radiation pulmonary fibrosis (RPF). Moreover, with the widespread utilization of immunotherapies such as immune checkpoint inhibitors as first- and second-line treatments for various cancers, the incidence of immunotherapy-related lung injury (IRLI), a severe immune-related adverse event (irAE), has rapidly increased. To date, we know relatively little about the underlying mechanisms and signaling pathways of these complications. A better understanding of the signaling pathways may facilitate the prevention of lung injury and exploration of potential therapeutic targets. Therefore, this review provides an overview of the signaling pathways of RILI and IRLI and focuses on their crosstalk in diverse signaling pathways as well as on possible mechanisms of adverse events resulting from combined radiotherapy and immunotherapy. Furthermore, this review proposes potential therapeutic targets and avenues of further research based on signaling pathways. Many new studies on pyroptosis have renewed appreciation for the value and importance of pyroptosis in lung injury. Therefore, the authors posit that pyroptosis may be the common downstream pathway of RILI and IRLI; discussion is also conducted regarding further perspectives on pyroptosis as a crucial signaling pathway in lung injury treatment.

## Introduction

Therapy for lung cancer risks producing adverse events, such as radiation-induced lung injury (RILI) and immunotherapy-related lung injury (IRLI). The incidence of RILI ranges from 5%-50% (1), but the pathogenesis and mechanisms of RILI and IRLI remain largely unclear. Compared to those of IRLI, the signaling pathways of RILI have been relatively well defined with continuous exploration and are summarized clearly in many reviews (2–6). However, IRLI-related signaling pathways, not to mention crosstalk between RILI and IRLI, have historically been largely underexplored. To date, there has been little discussion about crosstalk among these pathways. In addition, the combination of radiotherapy and immunotherapy shows a significant synergistic therapeutic effect in cancer (7–10). It is thus imperative to determine whether adverse events will happen and to identify the mechanisms of these adverse events arising from combination treatment with radiotherapy and immunotherapy.

Therefore, this review revisits previous works regarding the signaling pathways of RILI and IRLI and summarizes the potential crosstalk between RILI and IRLI. These signaling pathways have potential for clinical application as therapeutic targets. Moreover, in this review, the combination of radiotherapy and immunotherapy is considered and it is hypothesized that pyroptosis is likely a common downstream pathway of RILI and IRLI, a recognition that may facilitate and guide further research.

## Signaling Pathways of RILI and Targeted Therapies for RILI

RILI is a dose-limiting complication of radiotherapy for thoracic cancers and manifests as lung tissue damage, which is involved in acute radiation pneumonitis and chronic radiation pulmonary fibrosis. Many clinical trials and experiments in animal models have shown that a complex response after radiation leads to RILI (11–13); this response includes epithelial cells, endothelial cells, fibroblasts, extracellular matrix (ECM) molecules, and infiltrating immune cells (14). The primary initiation mechanisms are direct DNA damage and reactive oxygen species (ROS) generation, which then trigger intracellular signaling and lead to the release of various molecules and cytokines to promote inflammation and the immune response (15, 16). After irradiation, damage-associated molecular pattern (DAMP) molecules are released from cells, contributing to the recruitment of neutrophils, macrophages, leukocytes, and lymphocytes (17). With the transmigration of immune cells, numerous cytokines such as interleukin 3 (IL-3), interleukin 6 (IL-6), interferon-γ (IFN-γ), transforming growth factor β (TGF-β), tumor necrosis factor α (TNF-α) and high-mobility group box 1 (HMGB1) accumulate in impaired lung tissue. These events initiate an inflammatory response, leading to acute pneumonitis and chronic pulmonary fibrosis through diverse signaling pathways (18). In addition, hypoxia-inducible factors (HIFs) play a crucial role in the response to tumor reoxygenation and secondary hypoxic environments after the transmigration of immune cells (19, 20).

**Figure 1:**
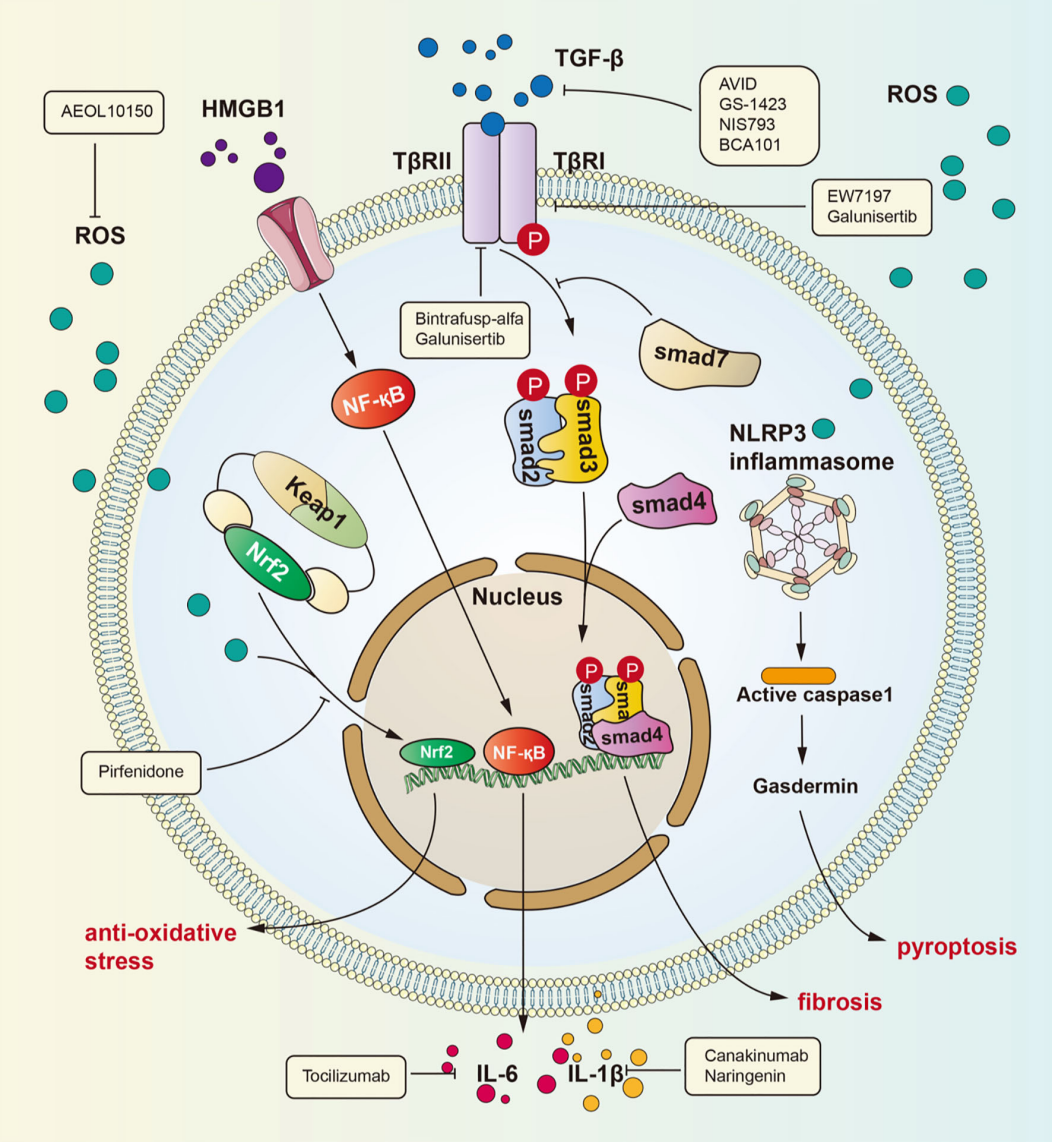
Signaling pathways for radiation-induced lung injury. Radiation induces lung injury *via* these ways showed in the figure. Activated HMGB1 binds to TLR4. It leads to NF-kB into nucleus and interaction with DNA, therefore promoting IL-1β and IL-6 expression which can cause lung inflammation. TGF-β can be activated by ROS. Activated TGF-β can bind to TGF-βRII therefore phosphorylating Smad2 and Smad3 which can form a complex with Smad4. The complex can regulate gene expression to promote fibrosis. ROS is produced after radiation and cause dissociation of NrF2 form complex. Dissociated NrF2 can regulate gene expression to suppress oxidative stress.

These diverse signaling pathways exert different effects in epithelial cells, endothelial cells, fibroblasts, extracellular matrix molecules, and infiltrating immune cells. Below, we list three main signaling pathways of RILI in **Figure 1**.

### TGF-β/Smad Signaling Pathway

Many investigations have identified TGF-β as the key molecule in many human fibrotic conditions, and TGF-β promotes the process of fibrogenesis in tissue cells and the extracellular matrix (21–23). It is currently well accepted that the TGF-β/small mother against decapentaplegic (Smad) pathway is a major signaling pathway leading to lung fibrosis resulting from many diseases (24). Among the TGF-β family members, TGF-β1 is considered the “master switch” for fibrosis (25, 26). The specific signaling pathway is as follows: TGF-β recognizes and binds transforming growth factor β receptor II (TGFβRII), which then phosphorylates transforming growth factor β receptor I (TGFβRI). After phosphorylation of TGFβRI, Smad2 and Smad3 are phosphorylated and form a heterotrimeric complex with Smad4 to translocate into the nucleus and regulate the expression of target genes and the transcription of profibrotic molecules, including α-smooth muscle actin (α-SMA), collagen I and tissue inhibitors of matrix metalloproteinases (TIMPs) (27). Then, these profibrotic molecules induce myofibroblast activation, matrix deposition, and epithelial-to-mesenchymal transition (EMT) to cause fibrosis. In addition, recent studies have identified the action of profibrotic microRNAs and long noncoding RNAs induced by the TGF-β/Smad signaling pathway in pulmonary fibrosis (28).

Roberts et al. found that Smad3 contributes to the pathogenic effects of TGF-β (29). This group observed that loss of Smad3 can block TGF-β-mediated epithelial-to-mesenchymal fibrosis and attenuate the development of fibrotic sequelae of ocular and renal injury in mice with targeted deletion of Smad3. Moreover, Cao and colleagues demonstrated that polydatin mitigates pulmonary fibrosis caused by irradiation by inhibiting the TGF-β/Smad signaling pathway and the EMT process (30). Park et al. identified that EW-7197, a novel small molecule inhibitor of TGF-β type I receptor kinase (ALK5), can inhibit pulmonary fibrosis by blocking the TGF-β/Smad signaling pathway and can decrease the expression of collagen, α-SMA, and fibronectin in mice (31). These studies confirm that the TGF-β/Smad signaling pathway is the crucial signaling pathway of RILI. Recently, new research has proven that RAS-responsive element binding protein 1 (RREB1) is a RAS-regulated Smad cofactor that drives the expression of profibrotic genes in EMT, which deepens our understanding of the association between the RAS and TGF-β pathways for the coordinated induction of EMT (32). Multiple approaches that interfere with the TGF-β/Smad signaling pathway have shown protective effects in preclinical models of pulmonary fibrosis (33). Therefore, further study of this pathway is expected to identify new therapeutic targets for pulmonary fibrosis. Indeed, many recent clinical trials have recently evaluated the anti-inflammatory and antifibrotic effects of TGF-β signaling pathway inhibition.

### HMGB1/TLR4 Signaling Pathway

HMGB1, an HMG protein, senses and coordinates the cellular stress response and functions as a damage-associated molecular pattern. HMGB1 exerts widespread biological effects throughout the body, including effects on metabolism, inflammation, immunity and cell death (34–36). In addition, many studies have verified that HMGB1 participates in lung inflammation and lung injury (37–39). The underlying mechanisms by which HMGB1 induces lung inflammation include several activated signaling pathways: ERK, JNK, PI3K/Akt, JAK and NF-κB (34). Although HMGB1 has been demonstrated to be involved in lung inflammation, its role in RILI has not been explicitly revealed. Generally, nuclear HMGB1 acts as a DNA chaperone involved in physiological processes such as DNA replication and transcriptional regulation, and extracellular HMGB1 actively secreted by immune cells or passively released by nonviable and injured cells regulates cell proliferation and inflammation by binding to multiple surface receptors, such as Toll-like receptor 2 (TLR2), Toll-like receptor 4 (TLR4), and receptor for advanced glycation end products (RAGE) (40). Reports indicate that blocking the HMGB1 signaling pathway can protect against early RILI and attenuate radiation-induced vascular injury, identifying a potential signaling pathway in RILI (41–43). Macrophages killed by high-dose irradiation can secrete HMGB1 to activate NF-κB through binding to TLR4, leading to an inflammatory response, and this process has been proven by Mei and colleagues (44, 45). Recently, a series of investigations have identified the role of the HMGB1/TLR4 signaling pathway in lung injury. Meng and coworkers identified the protective effect of dexmedetomidine in lung injury through the HMGB1-mediated TLR4/NF-κB pathway (46), and Liu et al. reported that fibroblast growth factor 10 exerts anti-inflammatory and cytoprotective effects to alleviate particulate matter-induced lung injury by inhibiting the HMGB1/TLR4 pathway (47). These studies suggested that HMGB1 plays an important role in mediating RILI and that the HMGB1/TLR4 pathway is a crucial signaling pathway that mediates RILI. Research has confirmed that HMGB1/TLR4 pathway activity induces RILI through downstream effectors such as NF-κB, JNK, and ERK1/2 and that this process can be inhibited by glycyrrhizin (48). The identification of the HMGB1/TLR4 signaling pathway elucidates the pathogenesis of RILI from a new perspective and identifies potential therapeutic targets for RILI.

### Nrf2/ARE Signaling Pathway

The nuclear factor erythroid 2 related factor 2 (Nrf2)/antioxidant response element (ARE) signaling pathway is one of the most important mechanisms in the body’s defense against oxidative stress. Nrf2 is an original member of the mammalian cap ‘n’ collar transcription factor family with a highly conserved basic region-leucine zipper (bZIP) motif (49), and contributes to the anti-inflammatory process and anti-oxidative stress response by orchestrating the recruitment of inflammatory cells and regulating gene expression through ARE (50–52). Under physiological conditions, Nrf2 is sequestered by Kelch-like ECH-associated protein 1 (Keap1) in the cytosol. However, under oxidative stress, such as that induced by radiation, Keap1 is modified and leads to the release of Nrf2, which translocates to the nucleus and activates ARE to express ARE-dependent genes for the oxidative stress response. Nrf2 contains three nuclear localization signals (NLSs), namely, NLS1, NLS2, and NLS3, which are critical for the nuclear import of Nrf2 (53). The translated gene products exert cytoprotective effects against ROS and include, including heme oxygenase-1 (HO-1), glutathione S-transferase (GSTs), the aryl hydrocarbon receptor (AhR), uridine 5’-diphospho-glucoronyl transferase (UGT), and sulfotransferases (SULTs) (51). For instance, HO-1 can catalyze the freeing of heme-bound Fe to generate biliverdin, and biliverdin can then be reduced to bilirubin with the generation of carbon monoxide to exert an anti-inflammatory effect (54). With radiotherapy-induced accumulation of ROS in lung tissue, the Nrf2/ARE signaling pathway plays a critical role in the maintenance of cellular homeostasis under oxidative stress (55–57).

Mathew and colleagues found using wild-type and genetically engineered mif (-/-) mice exposed to 20 Gy single-fraction thoracic radiation that migratory inhibition factor (MIF) may contribute to age-related susceptibility to thoracic radiation *via* Nrf2 (58). Similarly, Traver and coworkers found that loss of Nrf2 promotes loss of alveolar type 2 cells, whose injury initiates a fibrotic response, in different C57BL/6 mice exposed to a thoracic radiation dose of 12 Gy (59). However, the results of their investigations are consistent with those of a previous study verifying that Nrf2 deficiency reduces the life span of mice administered thoracic irradiation (60). Duru et al. confirmed the radioprotective role of Nrf2 in RILI *via* direct binding to the miR-140 promoter and elucidated the mechanism by which irradiation promotes Nrf2 nuclear translocation and subsequent activation of ARE-dependent genes (61). Furthermore, another investigation showed that Nrf2 deficiency exacerbates but Nrf2 overexpression significantly alleviates radiation-induced histopathological damage (62). All of these studies supported the protective role of Nrf2 in RILI, but the downstream mechanisms are largely unexplored. Therefore, focusing on the Nrf2/ARE signaling pathway may aid in the prevention and treatment of RILI.

In addition to the abovementioned signaling pathways, other possible pathways, such as melatonin-mediated miR-30e/nucleotide-binding domain-like receptor protein 3 (NLRP3), chemokine C-C motif ligand 2 (Ccl2), and Wnt/β-catenin signaling pathways, may play an important role in mediating RILI (63–65). Recently, research has also showed that ferroptosis inhibitors tremendously alleviate RILI, indicating the role of ferroptosis in RILI (66, 67). Similar to radiation-induced toxicity in head and neck cancers, single nucleotide polymorphisms of DNA repair and apoptosis genes may influence the severity of radiation-induced toxicity in RILI since data have confirmed involvement of ERCC1, ERCC5, TP53 and MDM2 in radiation-induced toxicity in head and neck cancer (68). Those results showed that relevant single nucleotide polymorphisms in DNA repair (ERCC1 and ERCC5) and apoptosis (MDM2 and TP53) genes might be linked to a higher risk of several grade 3-4 adverse effects including dermatitis, cervical skin fibrosis, xerostomia, and osteoradionecrosis. A profound recognition of signaling pathways underlying RILI would contribute to its prevention and control and provide potential therapeutic targets and strategies. Thus, much exploration and discovery is still needed to completely determine the signaling pathways of RILI.

Additionally, dosimetric factors play a critical role in RILI. There are few doubts that dosimetric parameters including irradiated volume, mean lung dose (MLD), dose delivered, schedule, and tumor location are risk factors for RILI (69, 70). Among these parameters, MLD is a key risk factor, and several studies have elucidated various cutoffs that associate with RILI (e.g. 16-18 Gy) (70, 71). Other studies have revealed associations with V20 and V30 (lung volume receiving 20 or 30 Gy, respectively) (72, 73), and V20-V40 for the postoperative setting (74).

In efforts to reduce dose exposure to by the normal lung, several new technologies have been recently developed. For instance, the magnetic resonance imaging linear accelerator (MR-Linac) provides more detailed real-time visualization of the tumor and surrounding tissue anatomy and facilitates the more precise use of adaptive re-planning (75). Additionally, proton beam therapy takes advantage of the unique biological properties of heavy ions (e.g. the proton), which deposits maximal dose at a certain point (the Bragg peak), distal to which there is a very sharp dropoff in dose including virtually no dose as the beam exits the patient (76, 77).

### Targeted Therapies for RILI

Many signaling pathways are activated under radiation stress, such as the main pathways introduced above. A common effect of these pathways is collagen aggregation. In the clinic, glucocorticoid drugs are the mainstay of RILI treatment. As the understanding of the mechanism of RILI has deepened, many drugs that can precisely target molecules in these signaling pathways or affect their common components have been identified. TGF-β is increasingly recognized for its role in the tumor environment. TGF-β not only can promote inflammation and fibrosis of the lung under radiation but also has an essential effect on tumor progression and escape (78). Many clinical trials have focused on inhibition of the TGF-β pathway to increase the curative effect and reduce side effects. There are four main types of drugs that inhibit the TGF-β pathway: anti-TGF-β antibodies, such as AVID200 (NCT03834662), GS-1423 (NCT03954704), NIS793 (NCT02947165), and BCA101 (NCT04429542); anti-TGF-β receptor antibodies, such as galunisertib (NCT02452008) and bintrafusp alfa (NCT04481256); small molecule inhibitors of TGF-β receptor serine/threonine kinase, such as SH3051 (NCT04423380); and anti-latent TGF-β monoclonal antibodies, such as SRK-181 (NCT04291079). Pirfenidone, an anti-inflammatory drug, is approved for use in patients with idiopathic pulmonary fibrosis (79–82). Pirfenidone can inhibit the TGF-β pathway to decrease radiation-induced tissue fibrosis (83). Researchers found that TGF-β release from M2 alveolar macrophages is decreased with pirfenidone treatment (84). In addition, pirfenidone affects the Nrf2 signaling pathway. One article reported that Nrf2 expression was upregulated in mice with lung fibrosis treated with pirfenidone, which revealed that pirfenidone may have an anti-oxidative effect (85). Glycyrrhizin can inhibit the HMGB1/TLR4 signaling pathway, therefore potentially attenuating RILI (48). Nintedanib, a kinase inhibitor that blocks VEGFR/PDGFR/FGFR, is another drug approved for idiopathic pulmonary fibrosis, and it can also be used to treat advanced non-small cell adenocarcinoma (86). It can also inhibit collagen aggregation, thereby ameliorating lung fibrosis (87). Some researchers have also found that nintedanib can inhibit fibrosis by means of suppressing activation of the TGF-β pathway (88, 89). However, the specific mechanisms are unclear. Endostar is an angiogenesis inhibitor that can attenuate liver fibrosis (90), and also can suppress scar formation in hypertrophic conditions by reducing VEGF expression (91, 92). Some researchers have explored whether Endostar can attenuate lung fibrosis after radiation therapy in non-small cell lung cancer (NSCLC) patients in clinical trials, although the results have not yet been reported. Enalapril, an angiotensin converting enzyme inhibitor, also plays an important antifibrotic role (93). Some researchers found that enalapril can suppress NF-kB signaling pathway activation in the context of pulmonary hypertension (94). The NF-kB pathway can promote fibrosis, as mentioned above. Further studies showed that enalapril can attenuate lung fibrosis caused by radiation (95). Next, AEOL10150 is an antioxidant that can catalyze ROS dismutation and alleviate ROS-mediated injury in rhesus macaques (96, 97). Tocilizumab, an anti-IL-6 receptor antibody, can reduce the inflammatory cytokine storm in patients with severe disease and increase mortality to some degree (98, 99). However, whether the drug has any effect on RILI remains to be studied. Canakinumab is an IL-1β antibody that has a potential effect on NSCLC progression and inflammation caused by IL-1β (100). Naringenin has also been proven to reduce RILI by decreasing the production of IL-1β (101).

## Signaling Pathways of IRLI and Treatment Thereof

Immunotherapy is a type of antineoplastic therapy based on mechanisms of the immune system aimed at facilitating immune function to kill cancer cells. Cancer immunotherapies can be divided into the following five classes: immune checkpoint inhibitors, lymphocyte-promoting cytokines, engineered T cells such as CAR T cells, agonistic antibodies against costimulatory receptors, and cancer vaccines. Among these classes, immune checkpoint inhibitors are the most commonly used (102). Many clinical trials of PD-1, PD-L1, and CTLA-4 inhibitors have been conducted. Nivolumab, pembrolizumab, durvalumab, and atezolizumab are the four major drugs approved by the FDA for the treatment of NSCLC patients. Although immune checkpoint inhibitors show a significant therapeutic effect in cancer, evidence has indicated the emergence of adverse events of these drugs, called immune-related adverse events (irAEs), as the number of patients exposed to these drugs has increased (103, 104). In addition, the safety and efficacy of immunotherapies and the management of irAEs have been widely discussed (105–108). Immune checkpoint inhibitors reactivate T cells to kill cancer cells but also destroy immune homeostasis, which has serious adverse impacts, including autoimmunity and nonspecific inflammation, on almost all bodily organs. It is estimated that irAEs occur predominantly in melanoma, lung, kidney, and other cancers (109, 110). Regarding IRLI, few studies and experiments have explored the related signaling pathways, and knowledge of this phenomenon is relatively superficial, although many case reports have referred to this complication caused by immune checkpoint inhibitors (111–113). Here, we summarize some possible mechanisms and present some recent advances in IRLI.

**Figure 2:**
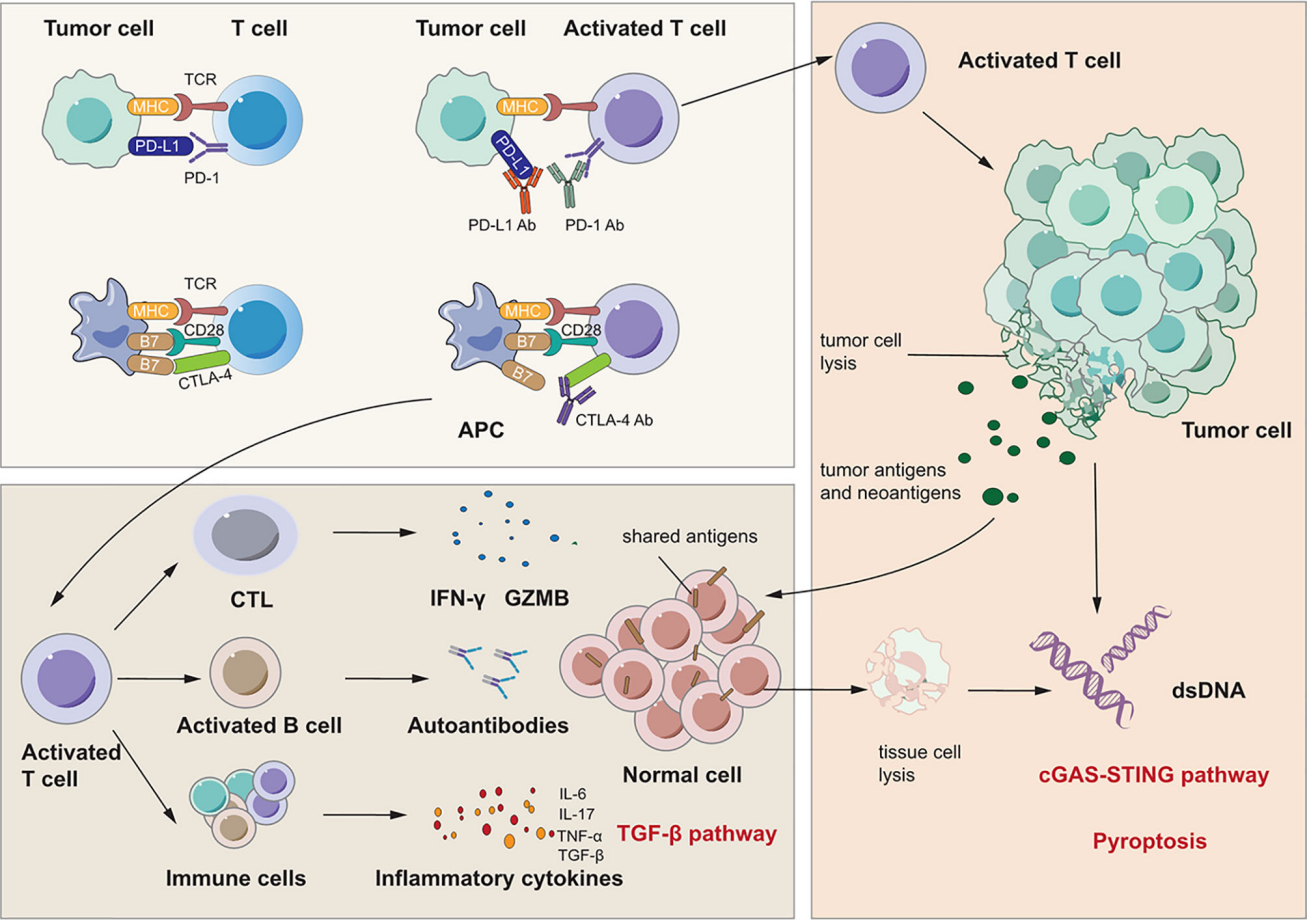
Signaling pathways for immunotherapy-related lung injury. Immune checkpoint inhibitors like PD-1 Ab, PD-L1 Ab and CTLA-4 Ab can bind to PD-1, PD-L1 and CTLA-4 specifically. This can activate immune cells like T cell and CTL to release many cytokines such as IFN-γ and IL-17 which can cause lung inflammation. At the same time, activated CTL can cause tumor cell lysis which releases tumor antigens and neoantigens. There are shared antigens in normal cells which can be recognized by T cells inducing tissue damage and inflammation. Besides, there are pre-existing antibodies in microenvironment with unlclear mechanisms which is related to irAEs.

There are several reviews about the mechanisms of irAEs that inspire discussion about signaling pathways in IRLI (114–120). Moreover, Zhai et al. suggested the risk factors for immune checkpoint inhibitor pneumonitis and explored potential mechanisms closely related to this complication (121). We summarize three proposed signaling pathways that may account for the immunopathogenesis of immune checkpoint inhibitor-induced lung injury: generalized immune activation owing to checkpoint neutralization, preexisting autoantibodies, and off-target effects of T cell-mediated immunity. These signaling pathways are shown in **Figure 2**.

First, immune checkpoint inhibitors promote a shift from the exhausted T cell phenotype to an active effector phenotype, and generalized immune activation facilitates the production of cytokines due to T cell activation (122, 123). Reactivated T cells may recognize shared antigens expressed in normal lung tissue, leading to cytotoxic activity. Suresh et al. observed that the alveolar immune cell landscape is dysregulated in checkpoint inhibitor pneumonitis and found increased numbers of BAL central memory T cells and decreased expression of CTLA-4 and PD-1 in BAL regulatory T cells (Tregs) (124). PD-1 and CTLA-4 on Tregs are critical targets of immunotherapy and have negative regulatory effects on immune cells (125, 126). Thus, these increases in activated T cells may induce inflammation and an immune response in lung tissue. In addition, cytokines released from these cells may participate in the process of lung injury and function as important biomarkers for irAEs (123, 127, 128). Activated Th1 and Th17 T lymphocytes may contribute to the production of proinflammatory cytokines such as IFN-γ and interleukin-17 (IL-17) (123, 129, 130). Moreover, a case report stated that the levels of C-reactive protein and IL-6 were elevated in a patient who developed immune checkpoint inhibitor pneumonitis after atezolizumab treatment (131). These cytokines may induce lung injury as they do in RILI, as discussed above. Second, preexisting autoantibodies seem to be a potential pathogenic mechanism in IRLI. Recent studies have reported that patients with disease-associated autoantibodies but not the corresponding clinical syndromes before checkpoint inhibitor therapy subsequently develop organ-specific irAEs (132,133). A new study proved that the presence of some preexisting antibodies was related to the development of irAEs in patients with NSCLC treated with nivolumab or pembrolizumab (134). However, the specific effect and mechanisms of these preexisting antibodies remain unclear. Third, neoantigens and tumor antigens are released upon CD8+ cytotoxic T lymphocyte-mediated cell lysis, a phenomenon termed epitope spreading. Reports indicate that reinvigoration of exhausted T cells can occur after epitope spreading, leading to autoantigen targeting, which contributes to myocarditis and pneumonitis (135, 136). These studies indicate that a similar mechanism may occur in other organs and play a vital role in IRLI.

In addition to these mechanisms that induce lung injury, other signaling pathways also contribute to IRLI. Recently, Jodai and coworkers reported the first case of acute eosinophilic pneumonia (AEP) as an irAE in a lung cancer patient who had received PD-1 blockade therapy (137). This study proposed that blockade of the PD-1-PD-L2 interaction can activate T helper 2 (Th2) cells and promote the release of cytokines that induce eosinophil transmigration from the bone marrow to lung tissue.

Similar to RILI as mentioned above, an increasing level of inflammatory cytokines including IL-3, -6, -10, and -17; TNF-α; and TGF-β leads to checkpoint inhibitor pneumonitis due to generalized immune activation (121). TGF-β, similar to the signaling pathway in RILI, recognizes and binds TGFβRII and then regulates the expression of target genes. Another important cytokine is IL-6, which is an important mediator of immune-related adverse events in non-small cell lung cancer patients treated with immune checkpoint blockade (131). IL-6 is believed to play a role in irAEs, especially since inhibition of IL-6 yields significant resolution of such symptoms (131, 138). ICIs cause aberrant activation of T cells and/or activation of tumor-reactive T cells against antigens that are shared by tumor and normal tissue. With the activation, proliferation and expansion of T cells, cytotoxic T cells may recognize self-antigens and directly attack normal cells *via* cytotoxic granules (perforin and granzymes), cytokines and Fas/FasL interactions while Th cells may secret IL-2, IL-6, IL-17, IFN-γ and TNF-α. Moreover, abnormal activation of B cells which may be activated by a T cell–independent mechanism can produce autoantibodies and induce antibody-dependent cell-mediated cytotoxicity. All of these three pathways can attack normal cells and cause cell death. Generally, the type of cell death caused by T and B cells is apoptosis which is regarded as silent cell death (139). The apoptotic cells are engulfed by phagocytes and this process called as efferocytosis produces anti-inflammatory cytokines including TGF-β which then activates TGF-β pathway. However, when the amount of apoptotic cells exceeds the capacity of the macrophages, apoptotic cells undergo secondary necrosis (140). And TNF-α also has the ability to induce apoptosis or necrosis and to induce necroptosis. In addition, granzyme A released from cytotoxic lymphocytes may cleave gasdermin B to trigger pyroptosis in target cells. After the complex cell death, danger associated molecular patterns and self-DNA from dead cells may be recognized by inflammasomes and cGAS which mediate pyroptosis and cGAS-STING pathway to induce a robust inflammatory response. Thus, these signaling pathways are all common pathways for both RILI and IRLI.

Although immunotherapy seems very promising, it can induce many adverse effects. Moreover, because the complex immune system is inadequately understood, much remains to be done to determine how to reach equilibrium in order to amplify antitumor effects and minimize side effects. The mainstay of treatment for IRLI is immunosuppressive drugs such as glucocorticoids, mycophenolate mofetil, and cytokine inhibitors. Glucocorticoids have well-rcecognized powerful well-rcecognized anti-inflammatory effects. Mycophenolate mofetil can be converted into mycophenolic acid in the body, and mycophenolic acid can act as an inhibitor of T and B lymphocyte proliferation, thereby reducing the immune response (141). First, clinicians should determine and evaluate the grade of pneumonitis and administer corresponding treatment. Patients with grades I and II disease can be given low-dose prednisone and continue immune checkpoint inhibitor therapy. However, for patients with grade III or IV disease, clinicians should discontinue immune checkpoint inhibitor therapy and closely observe their vital signs (142). Clinicians should ideally detect checkpoint inhibitor pneumonitis relatively soon and give patients steroid pulse therapy, which can result in a good prognosis. For patients who are insensitive to steroids, IL-1β inhibitors, IL-6 inhibitors, TNF-α inhibitors and antifibrotic drugs are possible treatments. Infliximab (an IL-6 inhibitor) can alleviate IRLI but in turn can cause other adverse effects (143, 144). Regarding safety, efficacy and adverse events, more clinical trials and mechanisms of combination treatment should be explored.

## Crosstalk Among Signaling Pathways in RILI and IRLI

**Figure 3:**
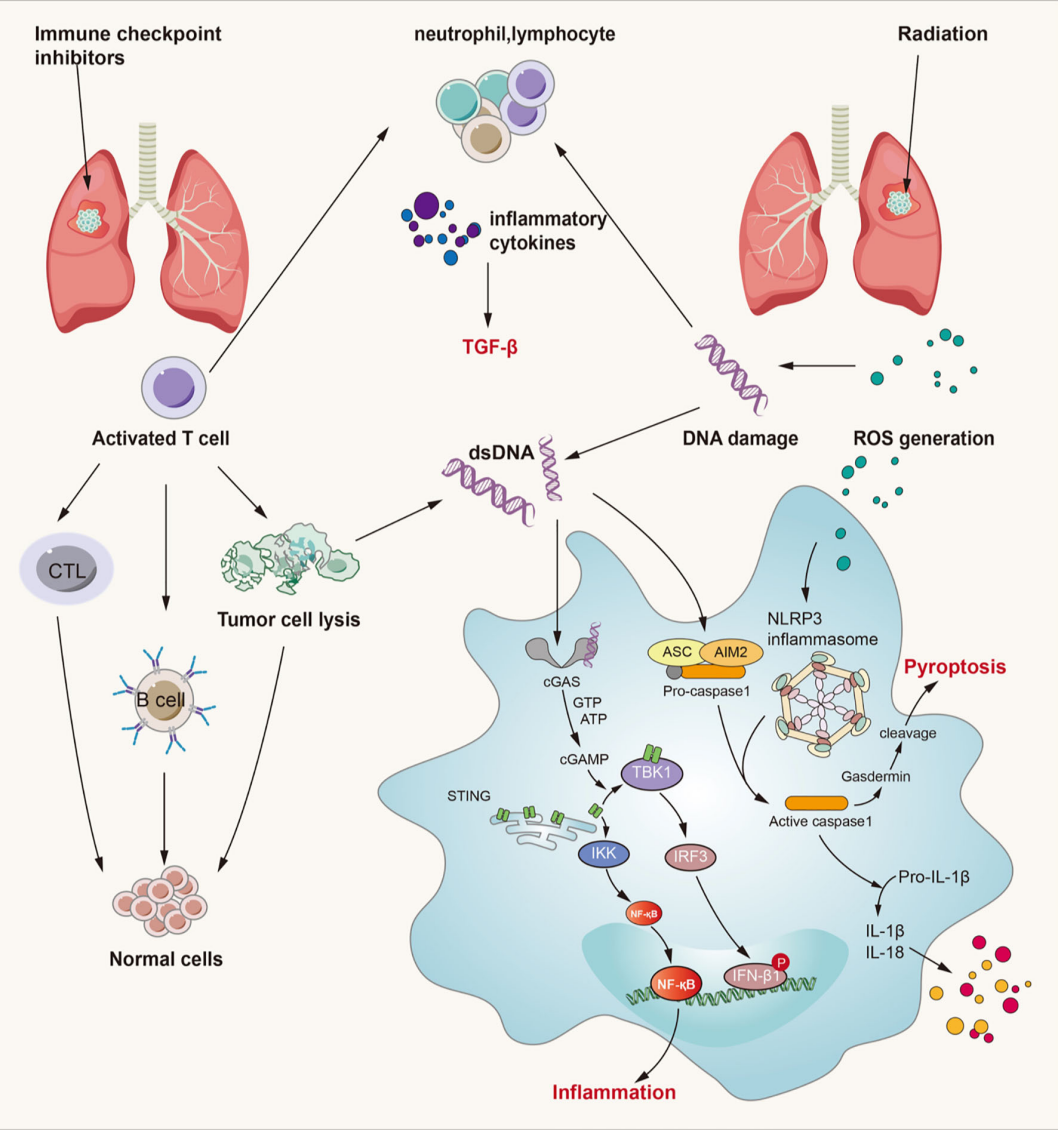
Crossed pathways for radiation-induced and immunotherapy-related lung injury. Radiation and checkpoint inhibitor therapy can induce immune cells activation. Activated cells can release many kinds of inflammatory factors like IL-3, IL-4, IL-10, IL-17, TNF-α, TGF-β and so on. Thereinto, TGF-β/Smad and cGAS-STING pathways play an important role in both RILI and IRLI. Except the classical function of TGF-β, it also exerts synergistic effects with PD-L1 in maintaining self-antigens tolerance and promoting Treg development. cGAS-STING signaling can recognize cytosolic DNA damaged by irradiation and CTL and then lead to pulmonary inflammation and fibrosis. ROS can lead to activation of NLRP3 inflammasome complex resulting in caspase-1 activation. Activated caspase-1 can induce IL-1β and IL-18 maturation which can lead to inflammation. At the same time, caspase-1 can cause gasdermin cleavage which can immediate pyroptosis.

On one hand, from the perspective of lung injury initiation, cell damage caused by radiotherapy and reactivation of T cells by immunotherapy contribute to a common endpoint event – the release of numerous cytokines. These cytokines can induce lung injury not only by direct damage to lung tissue through signaling pathways such as the TGF-β/Smad and TNF-α/NF-κB pathways, but also by indirect responses through recruitment of neutrophils, macrophages, and lymphocytes. Both RILI and IRLI induce the release of numerous cytokines from various cells to play different roles in damaged lung tissue. Among these cytokines, IL-4, -6, -10, and -17 have been demonstrated to be associated with radiation-related pneumonitis and the therapeutic effects of anti-PD-1/PD-L1 antibodies (145). In addition, some damage-associated signaling pathways, including the ROS/reactive nitrogen species (RNS) and cGMP–AMP synthase–stimulator of interferon genes (cGAS-STING) signaling pathways, participate in the initial process of lung injury (146–148). Considering the signaling pathways discussed above, it is obvious that cytokines are pivotal in mediating RILI and IRLI. We conclude that the possible crosstalk among signaling pathways mainly involves cytokines such as IL-3, -6, -10, and -17; TNF-α; and TGF-β. In addition, increasing importance has been attached to the role of TGF-β in the tumor microenvironment. Thus, strategies to inhibit the TGF-β pathway are considered essential components of future immunotherapy (78). The crosstalk among signaling pathways in RILI and IRLI remains largely unclear, but the mechanisms discussed above constitute the possible intersection. Here, we mainly discuss several signaling pathways closely related to radiation- or immune checkpoint inhibitor-induced lung injury from the perspective of initiation factors and manifestations of lung injury and we elaborate them in **Figure 3**.

### TGF-β Signaling Pathway

We have already mentioned TGF-β as a contributor to both RILI and IRLI, and the TGF-β/Smad signaling pathway as one of the most crucial pathways leading to pulmonary fibrosis. Moreover, TGF-β is not only the master regulator of fibrosis (27,149), but also plays a context-dependent role, and its biological effects are complex—it plays diverse roles in the regulation of cell proliferation and differentiation, wound healing, and the immune system, as well as key roles in pathologies, including fibrosis and cancer (150–152). The role of elevated plasma TGF-β during radiotherapy has long been recognized to predict a higher risk of developing pulmonary toxicity (153). After irradiation damage, TGF-β secreted by immune cells such as neutrophils, monocytes, and lymphocytes initiates a series of events leading to aseptic inflammation and pulmonary fibrosis; these events consist of increased expression of α-SMA, cell transformation into protomyofibroblasts and EMT. In addition, TGF-β plays a key regulatory role in immune responses and cancer progression (154–156), and it plays an undeniable role in cancer immunotherapy (78). Recently, Liu et al. confirmed that TGF-β suppresses Th2-cell-mediated cancer immunity, which promotes vessel remodeling, and that depletion of TGFBR2 in CD4+ T cells can suppress cancer progression *via* tissue healing and remodeling of the blood vasculature (157); thus, this group designed a strategy for targeted TGF-β signaling blockade in Th2 cells, which significantly inhibited tumor growth in mice (158). TGF-β signaling shows tremendous potential as an immunotherapy for the tumor microenvironment and has broad prospects in cancer treatment.

Here, we primarily discuss the TGF-β signaling pathway in immune checkpoint inhibitor-induced lung injury. With the use of immune checkpoint inhibitors, the T cell inhibitory signal is silenced, and activated T cells then stimulate generalized immune activation as well as the production of numerous cytokines, including TGF-β. In addition to the canonical TGF-β/Smad signaling pathway, TGF-β can also activate the MAPK, ERK, JNK, and PI3K pathways directly by ligand-bound receptors that modulate downstream cellular responses, including contributions to EMT, interactions with TGF-β/Smad signaling, or antagonism of Smad-mediated effects (159, 160). In addition, TGF-β is key to maintaining tolerance to self-antigens, and PD-L1 and TGF-β exhibit synergism in promoting Treg development (156). At sites of immune privilege or inflammation where TGF-β is present, PD-L1 may promote *de novo* generation of Tregs (161). Therefore, when we use PD-L1 blockade to treat cancer, the interaction between PD-L1 and TGF-β may be influenced, and the immune balance may be disrupted, damaging normal tissue. Therefore, the TGF-β signaling pathway may be one of the most important pathways in the crosstalk between RILI and IRLI.

### cGAS-STING Signaling Pathway

The link between recognition of microbial nucleic acids and activation of innate immune responses has been explored for several decades and was finally revealed to be the cGAS-STING pathway (162–164). In addition to mediating a protective immune response to infection, the cGAS-STING pathway can also detect tumor-driven DNA and impart antitumor immunity as well as play a role in the development of various inflammatory conditions (165–167). Under physiological conditions, cytosolic DNA is not present; thus, cGAS remains in an autoinhibitory state. However, under pathophysiological conditions, accumulated cytosolic DNA resulting from many harmful factors can be recognized by cGAS, which forms an oligomeric (2:2) complex with this DNA (168). DNA binding induces switch-like conformational changes in the activation loop of cGAS, which catalyzes the synthesis of 2’3’-cyclic GMP-AMP (cGAMP) from ATP and GTP (168, 169). 2’3’-cGAMP and other cyclic dinucleotides of bacterial origin can bind to STING and mediate the activation of IRF-3 by TANK-binding kinase 1 (TBK1), which translocates to the nucleus and initiates the transcription of the IFN-β gene (169, 170). In addition, STING activates NF-κB and induces the expression of inflammatory cytokines such as TNF, IL-1β and IL-6 in cooperation with TNF receptor-associated factor 6 (TRAF6), NF-κB essential modulator (NEMO), IKKβ, and TBK1 (162, 171–173).

We previously noted that a primary initiator of RILI is direct DNA damage, which then triggers intracellular signaling. Furthermore, mitochondrial DNA damage resulting from irradiation and ROS also provokes a series of inflammatory and immune responses and is more sensitive to the absence of repair mechanisms than is nuclear DNA damage (4). Since damaged DNA is released from nuclei or mitochondria, it is sensed by cGAS-STING signaling and leads to myriad biological consequences (162, 174). Mitochondrial damage and mtDNA have been reported to result in activation of the cGAS-STING pathway, leading to renal inflammation and fibrosis, which is similar to pulmonary inflammation and fibrosis (175). When radiation-induced DNA damage occurs, the DNA damage response (DDR) is activated depending on the context of damage and this is mediated by the central DDR kinases. There is an inextricable link between DDR signaling directly upstream of type I IFN signaling and it is shown that micronuclei link DNA damage by radiotherapy and/or DDR inhibitors to cytoplasmic nucleic acid sensors and type I IFN signaling (176, 177). Loss of function or inhibitors against numerous key DDR kinases have been shown to induce type I interferon production (178). Thus, DDR inhibitors can not only inhibit DNA repair and increase the sensitivity of radiotherapy, but also increase the number of micronucleus and small fragments of DNA in tumor cells induced by radiotherapy, and further activate the cGAS-STING pathway and the expression of type 1 IFN. As mentioned before, Borchiellini et al. have confirmed that relevant single nucleotide polymorphisms in DNA repair (ERCC1 and ERCC5) genes might influence the severity of radiation-related side-effects in HNSCC patients and others have verified that the inhibition of checkpoint kinase 1 and 2 (which activate cell-cycle checkpoints and serve a critical role in the DDR reduced NOTCH signaling) enhances the *in vitro* and *in vivo* response of HNSCCs to radiation (68, 179). These results all support the potential role of DDR inhibitors in radiation-induced toxicity and combination with RT. Additionally, based on cancer-associated DDR defects, DDR inhibitors as tumor-selective radiosensitizers may allow improved tumor control without increased normal tissue toxicity which reduces the incidence of RILI. In addition, the role of cGAS-STING signaling in lung inflammation and lung fibrotic diseases has been elucidated (147). Moreover, the cGAS-STING signaling pathway is closely related to natural antitumor immunity as well as immune checkpoint inhibitor therapy, which implies the potential of targeting this pathway to mediate IRLI (180, 181). When immune checkpoint inhibitors silence inhibitory signals for T cell activation and activate an effective antitumor response, accumulation of self-DNA released from dying tumor cells triggers the cGAS-STING pathway to induce the production of interferons and inflammatory cytokines (182). Activation of the cGAS-STING pathway can not only mediate innate antitumor immunity and produce synergistic antitumor effects with immune checkpoint inhibitors but also can elicit a robust inflammatory response by activating NF-κB and stimulating the production of inflammatory cytokines such as TNF, IL-1β, and IL-6 (181, 183). However, few investigations on the cGAS-STING pathway in RILI and IRLI have been conducted; thus, more research is needed to explore the role of cGAS-STING in lung injury.

### Pyroptosis

Pyroptosis is defined as a form of regulated cell death (RCD) that critically depends on the formation of plasma membrane pores by members of the gasdermin protein family, often (but not always) as a consequence of inflammatory caspase activation (184, 185). It occurs widely in all kinds of cells and leads to an inflammatory cascade response through the release of IL-1β and IL-18 (unlike the immune-silencing effect of apoptosis) (186, 187). Cheng et al. found that caspase-11-mediated endothelial pyroptosis underlies endotoxemia-induced lung injury (188), and several studies have addressed NLRP3 inflammasome- and AIM2 inflammasome-mediated pyroptosis in lung injury (189–192). Lung injury, especially pneumonitis, occurs more often as a result of the inflammatory response due to radiotherapy or immunotherapy. Therefore, pyroptosis may likely be the common downstream signaling pathway of RILI and IRLI.

A considerable number of studies have addressed the key role of pyroptosis in lung injury (189–191, 193, 194). Accumulating evidence suggests that NLRP3 inflammasome- and AIM2 inflammasome-mediated pyroptosis in both macrophages and epithelial cells plays a critical role in the development of radiation-induced tissue injury (195, 196). Generally, double-stranded DNA breaks caused by ionizing radiation can be recognized by the AIM2 inflammasome, which then activates the process of pyroptosis (197, 198). AIM2 translocates into the nucleus, localizes at the foci of double-stranded DNA breaks, and recruits ASC, which leads to caspase-1 activation. The active caspase-1 induces pore formation on the plasma membrane through the cleavage of gasdermin D. With the cleavage of gasdermin D, the enzymolysis and release of IL-1β and IL-18 lead to inflammation around the site. Otherwise, radiation-induced pyroptosis in bone marrow-derived macrophages mediated by the NLRP3 inflammasome is initiated by the recognizing of NLRs to PAMPs and DAMPs, and the downstream process is as described above (196, 199). Based on the evidence, a new study has showed that inhibition of AIM2 inflammasome-mediated pyroptosis by andrographolide contributes to amelioration of radiation-induced lung inflammation and fibrosis, further confirming the important role of pyroptosis in RILI (192). They found that andrographolide effectively prevented AIM2 from translocating into the nucleus to sense DNA damage induced by radiation in bone marrow-derived macrophages. Moreover, Wu and coworkers verified the effect of 5-androstenediol to prevent radiation injury in mice though inhibiting radiation-induced activation of caspase-1 and GSDMD by decreasing the interaction between AIM2 and ASC, which indicates that the AIM2 inflammasome is a key signaling pathway in radiation-induced lung injury (200). Han and colleagues preliminary confirmed that NLRP3 inflammasome induces pyroptosis in mice with radiation-induced lung injury though the detection of a series of pyroptosis related molecules (201).

Pyroptosis is also closely related to IRLI. As mentioned above, normal lung tissue cells are attacked and damaged due to generalized immune activation from checkpoint neutralization, preexisting autoantibodies, and off-target effects of T cell-mediated immunity. With lysis of these (along with neoplastic) cells, double-stranded DNA and endogenous danger signals may be recognized by the AIM2 inflammasome and DAMPs may be recognized by the NLRP3 inflammasome. This may mediate pyroptosis of tissue cells leading to pneumonitis in IRLI, similar to the events of pyroptosis in RILI. IL-1β and IL-18, as the products of inflammasome activation, result in proinflammatory T cell differentiation and target organ damage (186, 202). Moreover, PD-L1 in the nucleus may be closely associated with pyroptosis in tumor cells. A recent study proved that under hypoxic conditions, PD-L1-mediated gasdermin C expression can switch apoptosis to pyroptosis in cancer cells (203). This research shows a non-immune checkpoint function of PD-L1 and indicates that pyroptosis is likely the key signaling pathway in immunotherapy, inflammation, and tumor necrosis. Moreover, diversification of cell death has been valued for the interaction between antitumor immunity and distinct cell death mechanisms (204, 205). Wang et al. revealed the antitumor immune function of pyroptosis through a bioorthogonal system, finding that pyroptosis-induced inflammation contributes to robust antitumor immunity and can synergize with immune checkpoint blockade (206). Another study showed that gasdermin E can suppress tumor growth by granzyme B in killer cells, triggering caspase-independent pyroptosis in cancer cells (207). Similarly, Zhou et al. found that granzyme A released from cytotoxic lymphocytes cleaves gasdermin B to trigger pyroptosis in target cells and that PD-1/PD-L1 blockade can activate T cells to promote gasdermin B-mediated pyroptosis to kill cancer cells (208). However, in this process, the inflammatory response may be enhanced with the release of IL-1β and IL-18, and pyroptosis of cancer cells may lead to possible injury to normal cells in the tissue. On the other hand, Yang and coworkers attempted to summarize the role of inflammasomes on innate immunity and the natural history of autoimmune diseases. They showed that inflammasomes play a key role in lowering the threshold of immunity, thereby potentiating a number of autoimmune diseases (209). Si Ming Man et al. elaborated on the role of AIM2 inflammasomes in cancer and autoimmunity, that inappropriate recognition of cytoplasmic self-DNA by AIM2 contributes to the development of psoriasis, dermatitis, arthritis, and other autoimmune and/or inflammatory diseases (210). These investigations indicate the potential relationship between pyroptosis and autoimmunity, which is similar to the postulated mechanisms of IRLI. Nevertheless, there remains a lack of robust evidence to support the mechanism of pyroptosis in IRLI, and this area needs to be further explored.

## Conclusions and Further Prospects

Unfortunately, adverse events such as RILI and IRLI are not uncommon. More research and knowledge on the mechanisms of RILI and IRLI should be applied for the diagnosis and treatment of patients. Thus, more exploration and discovery to pinpoint the signaling pathways and mechanisms of RILI and IRLI are required. This review sheds new light on the crosstalk among these signaling pathways, which may provide targets for the prevention and treatment of RILI and IRLI. Furthermore, pyroptosis, a focus of frontier research, could be the key to elucidating mechanisms and solving problems including cancer and adverse events caused by cancer treatment.

## Author Contributions

JY, DC, and MW contributed to the study design, manuscript discussion and critical revision. ZZ and JZ drafted the manuscript. XL assisted with the collection and visualization of the data. JY, DC and VV revised and polished the manuscript. All authors contributed to the article and approved the submitted version.

## Funding

The study was supported by funds from the Academic Promotion Program of Shandong First Medical University (2019ZL002) and the foundation of National Natural Science Foundation of China (81972863, 8217102837 and 82030082) and Science Foundation of Shandong (ZR2020LZL016, ZR2021YQ52).

## Conflict of Interest

The authors declare that the research was conducted in the absence of any commercial or financial relationships that could be construed as a potential conflict of interest.

## Publisher’s Note

All claims expressed in this article are solely those of the authors and do not necessarily represent those of their affiliated organizations, or those of the publisher, the editors and the reviewers. Any product that may be evaluated in this article, or claim that may be made by its manufacturer, is not guaranteed or endorsed by the publisher.

## Glossary

**Table:**

RILI	Radiation-induced lung injury
RT	Radiotherapy
RP	Radiation pneumonitis
RPF	Radiation pulmonary fibrosis
irAEs	Immune-related adverse events
ROS	Reactive oxygen species
DAMPs	Damage associated molecular pattern molecules
IL-3	Interleukin 6
IL-6	Interleukin 6
IFN-γ	Interferon-γ
TGF-β	Transforming growth factor β
TNF-α	Tumor necrosis factor α
HMGB1	High-mobility group box 1
HIFs	Hypoxia-inducible factors
ECM	Extracellular matrix
Smad	Small mother against decapentaplegic
TGFβRII	Transforming growth factor β receptor II
TGFβRI	Transforming growth factor β receptor I
α-SMA	α-smooth muscle actin
RREB1	RAS-responsive element binding protein 1
EMT	Epithelial-to-mesenchymal transition
HMG	High-mobility group
ERK	Extracellular signal-regulated kinase
JNK	C-Jun N-terminal kinase
PI3K	Phosphatidylinositol 3-kinase
JAK	Janus kinase
NF-κB	Nuclear factor-kappaB
TLR4	Toll like receptor 4
Nrf2	Nuclear factor erythroid 2 related factor 2
ARE	Antioxidant response element
Keap1	Kelch-like ECH-associated protein 1
NLRP3	Nucleotide-binding domain-like receptor protein 3
Ccl2	Chemokine C-C motif ligand 2
CAR T	Chimeric antigen receptor T cells
CTLA-4	Cytotoxic T-lymphocyte-associated antigen 4
PD-1	Programmed cell death protein 1
PD-L1	Programmed death-ligand 1
PD-L2	Programmed death-ligand 2
BAL	Bronchoalveolar lavage
AIM	Absent in melanoma
Tregs	Regulatory T cells
Th1	T-helper 1
Th17	T-helper 17
NSCLC	Non-small cell lung cancer
AEP	Acute eosinophilic pneumonia
cGAS-STING	cGMP–AMP synthase–stimulator of interferon genes
RRP	Radiation recall pneumonitis
RCD	Regulated cell death
VEGFR	Vascular endothelial growth factor receptor
PDGFR	Platelet-derived growth factor receptor
FGFR	Fibroblast growth factor receptor
